# Predictive potential of macrophage migration inhibitory factor (MIF) in patients with heart failure with preserved ejection fraction (HFpEF)

**DOI:** 10.1186/s40001-018-0321-1

**Published:** 2018-05-04

**Authors:** Peter Luedike, Georgios Alatzides, Maria Papathanasiou, Martin Heisler, Julia Pohl, Nils Lehmann, Tienush Rassaf

**Affiliations:** 1 Department of Cardiology and Vascular Medicine, West German Heart and Vascular Center, University Hospital Essen, Hufelandstrasse 55, 45122 Essen, Germany; 2 Institute for Medical Informatics, Biometry and Epidemiology, University of Duisburg-Essen, Hufelandstr. 55, 45122 Essen, Germany

## Abstract

**Background:**

Prognostication in heart failure with preserved ejection fraction (HFpEF) is challenging and novel biomarkers are urgently needed. Macrophage migration inhibitory factor (MIF) is a pro-inflammatory cytokine that plays a crucial role in cardiovascular and various inflammatory diseases. Whether MIF is involved in HFpEF is unknown.

**Methods and results:**

Sixty-two patients with HFpEF were enrolled and followed up for 180 days. MIF plasma levels as well as natriuretic peptide (NP) levels were assessed. High MIF levels significantly predicted the combined end-point of all-cause death or hospitalization at 180 days in the univariate analysis (HR 2.41, 95% CI 1.12–5.19, *p* = 0.025) and after adjustment for relevant covariates in a Cox proportional hazard regression model (HR 2.35, 95% CI 1.05–5.27, *p* = 0.0374). Furthermore, MIF levels above the median were associated with higher pulmonary artery systolic pressure (PASP) as assessed by echocardiography (PASP 31 mmHg vs 48 mmHg in the low- and high-MIF group, respectively, *p* = 0.017). NPs significantly correlated with MIF in HFpEF patients (BNP *p* = 0.011; *r* = 0.32; NT-proBNP *p* = 0.027; *r* = 0.28).

**Conclusion:**

MIF was associated with clinical outcomes and might be involved in the pathophysiology of pulmonary hypertension in patients with HFpEF. These first data on MIF in HFpEF should stimulate further research to elucidate the role of this cytokine in heart failure.

*Trial registration* NCT03232671

## Background

Patients with signs and symptoms of heart failure and a normal left ventricular ejection fraction (LVEF) are said to have heart failure with preserved ejection fraction (HFpEF) [1]. HFpEF has a significant global economic burden due to increasing rates of hospitalization and mortality especially in the elderly population [2]. To date, HFpEF constitutes a complex of various symptoms, predominantly dyspnea, and fatigue, rather than a well-defined disease. Many questions about the underlying characteristics, pathophysiology, and treatment of HFpEF are not answered yet and recent guidelines of the European Cardiac Society (ESC) and the American Heart Association (AHA) emphasized the need for new biomarkers in the prevention, assessment, and management in the field [3, 4]. In contrast to heart failure with reduced ejection fraction (HFrEF), where the reduction in cardiac output is the driving force for disease progression, it is unclear in HFpEF if the abnormal myocardial relaxation is the origin or rather the common pathologic final path of a myriad of long lasting diseases like hypertension, diabetes, or chronic kidney disease [5, 6].

The diagnosis of HFpEF relies on four criteria according to the current ESC guidelines [4]. These are typical symptoms and/or signs of heart failure, an LVEF of > 50%, elevated levels of natriuretic peptides (NPs) (BNP > 35 pg/ml; NT-proBNP > 125 pg/ml), and additional evidence of structural heart disease or a diastolic dysfunction [4]. Despite being mandatory for the diagnosis, the exact role of NPs in clinical phenotyping in HFpEF has been less studied. This is partly because of inconsistencies related to poor test characteristics in this population and the fact that end-diastolic wall stress, the trigger for BNP/NT-proBNP release, has been found to be lower in HFpEF than in HFrEF [7, 8]. Moreover, HFpEF is a heterogenous clinical syndrome that is characterized by cardiovascular, metabolic, and pro-inflammatory diseases and thus cannot be simplified on impaired diastolic filling and increased wall stress. These uncertainties on the role of NPs in the pathophysiology of HFpEF raise the need for a panel of biomarkers that would reflect all the pathophysiological changes that take place in the development and disease course.

With regard to diagnostic markers that might reflect changes in metabolic, inflammatory, and cardiovascular diseases, the cytokine macrophage migration inhibitory factor (MIF) plays an emerging role. MIF is quasi-ubiquitously expressed and stored in numerous cell types, while specifically secreted from the pituitary gland upon endotoxaemia [9], from immune cells upon inflammatory stimulation, as well as from selected endothelial and parenchymal cells upon hypoxic, hyperoxic, and other stress stimuli [10]. MIF is a well-established mediator of a number of acute and chronic inflammatory diseases including atherosclerosis, chronic kidney disease, organ fibrosis, and rheumatoid arthritis [11–13]. MIF’s role in cardiovascular disease is dual, as it also has a clear-cut cardioprotective role in the setting of myocardial ischemia and reperfusion (I/R) injury, contrasting the bonafide negative function in the promotion of arteriosclerosis development [14]. The majority of experimental reports on the effect of MIF during myocardial I/R injury demonstrate an overall cardioprotective effect in the early reperfusion period of 24 h, whereas others report cardio depressant effects in later stages [14, 15].

MIF can be easily and robustly measured in the circulation of patients by applying a recently established protocol for accurate assessment [16]. Clinical observations showed that increased MIF plasma levels are closely associated with myocardial infarction, critical illness, rheumatoid arthritis, or chronic kidney disease [17–19]. Since MIF has both pro-inflammatory as well as oxidoreductase properties, it seems to be an ideal candidate to reflect the biological pathways involved in the pathophysiology that takes place in the development of HFpEF.

With regard to the need of deeper insight into the field of HFpEF, we here aimed to investigate the role of MIF and to assess its diagnostic and prognostic potential.

## Patients and methods

### Study setting and population

We conducted a prospective cohort study at the Department of Cardiology and Vascular Medicine at the West German Heart and Vascular Center, University Hospital Essen, Germany, a level III maximum care hospital. Over a period of 6 months *n* = 62 patients presenting with HFpEF were enrolled and MIF plasma levels and NP levels (BNP and NT-proBNP) were assessed. Follow-up (FU) was prospectively scheduled at 180 days. All patients underwent echocardiographic assessment of LVEF and structural parameters to justify the diagnosis of HFpEF according to current ESC guidelines. Patients presenting with symptoms of heart failure were only included if they fulfilled the criteria of HFpEF according to the current definition of the ESC [4]. Written informed consent was obtained from all patients. The study protocol was approved by the local ethics committee (ethics committee of the University Hospital Essen, Germany). The study was registered at clinicaltrials.org (NCT03232671).

### MIF measurements

Blood samples for determining MIF plasma levels were taken at the day of admission and immediately centrifuged at 1000×*g* for 15 min at 4 °C. Plasma was obtained and frozen at − 20 °C until measurement. MIF levels were determined using an enzyme-linked immunosorbent assay (ELISA, R&D, USA) as described previously [16, 18–20].

### Statistical analyses

Continuous variables are summarized as means (standard deviations) in case of normal distribution, otherwise as medians (interquartile ranges, IQR) and categorical variables as counts (percentages). Continuous data were evaluated for normality of distribution using the Kolmogorov–Smirnov test and by inspection of the histograms. Baseline MIF values were categorized to high- and low-MIF category based on the median value for the purpose descriptive analysis. Differences in continuous variables across the two MIF groups were tested with *t *test or the Mann–Whitney *U* test. The Chi-square test and Fisher’s exact test were used for testing association between categorical variables and MIF groups. Correlation of MIF with other biomarkers was evaluated by the Spearman’s rank-order correlation. The predictive accuracy of MIF for the combined end-point of all-cause death or hospitalization was evaluated by receiver operating characteristic (ROC) curve analysis. Kaplan–Meier analysis was conducted to estimate survival. The log-rank test was performed to determine differences across the MIF groups. Risk factors for death were assessed by Cox proportional hazards regression analysis. Variables entered the model based on clinical criteria and if previously significant in univariate analysis. The level of significance was set to 0.05. All analyses were performed using SPSS (IBM Corp., SPSS Statistics, Version 23.0. Armonk, NY).

## Results

### Patients’ characteristics

We enrolled consecutive patients presenting at the heart failure unit of the West German Heart and Vascular Center with symptoms and signs of heart failure and the proven diagnosis of HFpEF between April and October 2016. Seventy-one appropriate candidates were screened of which nine denied to participate. Of 62 participants, none was lost in the 180-day FU period. At 180-day FU, we reported 16.1% death due to any reason (10/62), 33.9% hospitalization due to any reason (21/62), and 46.8% for the combined end-point of death or hospitalization (29/62). The median MIF plasma level was 51.6 ng/mL (IQR 35.6 ng/mL) with a range from 6.4 to 168.6 ng/mL. There was a significant association between higher MIF levels and age in our study population (71.0 IQR 23.0 low MIF vs. 77.0 IQR 22.0 high MIF) (Table 1, *p* = 0.026) with higher MIF levels in the elderly participants. Patients with higher MIF levels were more likely to have worse NYHA functional class (*p* < 0.001), increased right ventricular load as documented by estimated pulmonary artery systolic pressure (PASP) (*p* = 0.0017), and more congestion as shown by the higher NT-proBNP (*p* < 0.005) and BNP levels (*p* = 0.0014). Higher MIF was associated with higher lactate dehydrogenase (LDH) levels which serve as a parameter for chronic tissue damage in patients with chronic diseases (*p* = 0.038). The distribution of comorbidities was equal in the high-MIF and low-MIF group and represented the typical pattern of HFpEF patients with a high proportion of atrial fibrillation, hypertension, and chronic kidney disease.

**Table 1:** Patient characteristics in the high-MIF and the low-MIF group

	Entire cohort, *n* = 62	MIF ≤ 51.58 ng/ml, *n* = 31	MIF > 51.58 ng/ml, *n* = 31	*p* value
Clinical data				
Age, years^a^	73.5 (21.0)	71.0 (23.0)	77.0 (22.0)	0.026
Male gender	26 (41.9)	14 (45.2)	12 (38.7)	0.80
BMI (kg/m^2^)	27.9 (4.6)	28.3 (4.9)	27.5 (4.3)	0.47
SBP (mmHg)^a^	132.5 (23.8)	132.0 (24.0)	133.0 (32.0)	0.21
DBP (mmHg)	72.5 (13.5)	73.9 (13.4)	71.0 (13.7)	0.48
Dyspnea NYHA III/IV	36 (58.1)	11 (35.5)	25 (80.6)	< 0.001
Comorbidities				
AF	32 (51.6)	13 (41.9)	19 (61.3)	0.13
CHD	34 (54.8)	13 (41.9)	21 (67.7)	0.041
Hypertension	48 (77.4)	23 (74.2)	25 (80.6)	0.54
Diabetes mellitus	16 (25.8)	6 (19.4)	10 (32.3)	0.25
CKD	24 (38.7)	13 (41.9)	11 (35.5)	0.60
COPD	11 (17.7)	3 (9.7)	8 (25.8)	0.10
Medication				
Betablocker	51 (82.3	26 (83.9)	25 (80.6)	0.74
MRA	17 (27.4)	10 (32.3)	7 (22.6)	0.39
ACE inhibitor	33 (53.2)	19 (61.3)	14 (45.2)	0.20
Diuretics	45 (72.6)	21 (67.7)	24 (77.4)	0.39
Oral anticoagulants	30 (48.4)	12 (38.7)	18 (58.1)	0.13
Echocardiographic parameters				
EF (%)	56.5 (5.5)	56.3 (5.7)	56.7 (5.5)	0.78
LVEDD, cm	4.9 (0.8)	5 (0.6)	4.8 (0.9)	0.27
PASP (mmHg)^a^	36.0 (22.8)	31.0 (20.0)	48.0 (20.0)	0.0017
LAA (cm^2^)	24.4 (7.2)	22.6 (5.2)	26.2 (8.6)	0.06
Laboratory values				
WBC (cells/nl)^a^	6.7 (2.8)	6.2 (2.4)	7.1 (2.3)	0.11
Hemoglobin (g/dl)	12.3 (1.5)	12.6 (1.3)	12.4 (1.8)	0.59
Creatinine (mg/dl)^a^	1.2 (0.5)	1.2 (0.7)	1.2 (0.4)	0.65
Urea (mg/dl)^a^	21.0 (11.0)	20.0 (12.0)	22.0 (10.0)	1.00
LDH (U/l)^a^	248.0 (88.5)	236.0 (70.0)	261.0 (119.0)	0.038
CRP (mg/dl)^a^	0.49 (1.2)	0.49 (0.51)	0.49 (1.2)	0.25
BNP (pg/ml)^a^	229.8 (284.0)	134.4 (246.1)	298 (367.8)	0.0014
NT-proBNP (pg/ml)^a^	1329.0 (3608.8)	612.0 (1397.0)	2517.0 (3644.0)	0.005

*BMI* body mass index, *SBP* systolic blood pressure, *DBP* diastolic blood pressure, *NYHA* New York Heart Association, *AF* atrial fibrillation, *CHD* coronary heart disease, *CKD* chronic kidney disease, *COPD* chronic obstructive pulmonary disease, *MRA* mineralocorticoid receptor antagonist, *ACEI* angiotensin converting enzyme inhibitor, *EF* ejection fraction, *LVEDD* left ventricular end-diastolic diameter, *PASP* pulmonary artery systolic pressure, *LAA* left atrial area, *WBC* white blood cells, *LDH* lactate dehydrogenase, *CRP* c-reactive protein, *BNP* b-type natriuretic peptide, *NT-proBNP* amino-terminal pro-b-type natriuretic peptide

^a^Values represent median (interquartile range)

### High MIF plasma levels predict the combined end-point of 180-day mortality or hospitalization

**Fig. 1:**
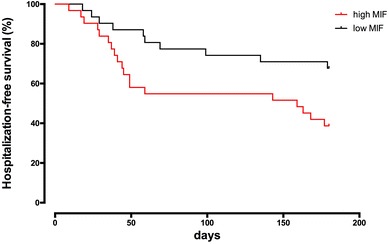
Kaplan–Meier curves for all-cause death or hospitalization at 180 days by low (≤ 51.58 ng/ml) vs. high (> 51.58 ng/ml) MIF category. Log-rank *p* = 0.020

**Fig. 2:**
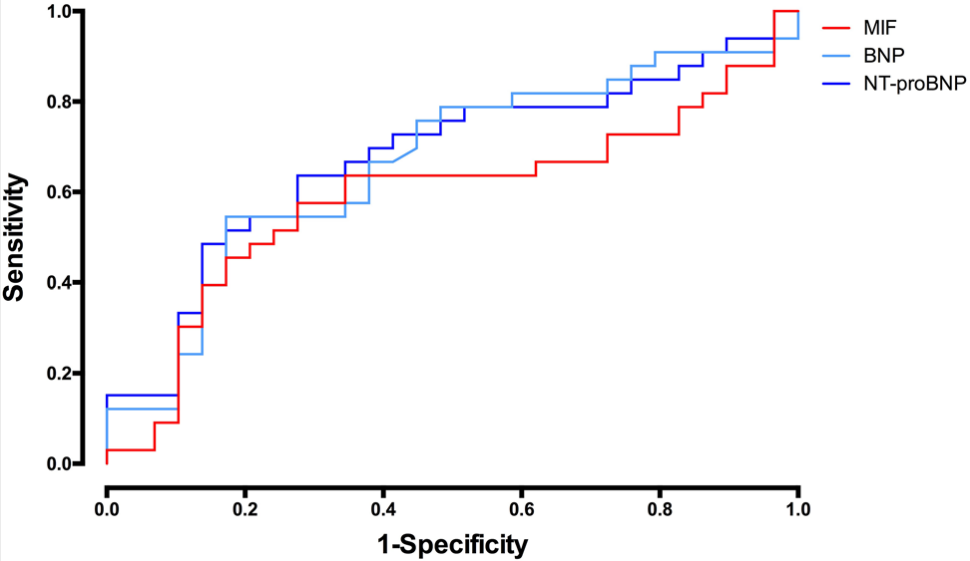
Receiver operating characteristic curves for all-cause death or hospitalization at 180 days. AUC-MIF: 0.59, *p* = 0.23, AUC-BNP: 0.66, *p* = 0.027, AUC-NT-proBNP: 0.68, *p* = 0.017

Kaplan–Meier analysis revealed an increased event rate in the high-MIF group (Log-rank = 0.020, Fig. 1). This association of high MIF levels with all-cause death or hospitalization was tested in a Cox proportional hazards regression analysis (Table 2). After adjustment for several covariates (age, gender, CHD, NT-proBNP), MIF level above the median remained statistically significant (adjusted HR 2.35, 95% CI 1.05–5.27, *p* = 0.0374) (Table 2). The predictive accuracy of MIF for the occurrence of death or hospitalization at 180 days was comparable but inferior to that of NPs as shown by the ROC curves (BNP AUC 0.66, *p* = 0.027; NT-proBNP AUC 0.68, *p* = 0.017) (Fig. 2).

**Table 2:** Association of MIF with time-to-event (all-cause death or hospitalization)

	HR (95% CI)^a^	*p* value
Unadjusted	2.41 (1.12–5.19)	0.025
Model 1	2.42 (1.09–5.37)	0.030
Model 2	2.35 (1.05–5.27)	0.037

Model 1: Adjusted for age, gender, CHD

Model 2: Adjusted for age, gender, CHD, NT-proBNP

Cox proportional hazards regression analysis adjusted for known risk factors. *CHD* coronary heart disease, *NT-proBNP* amino-terminal pro-b-type natriuretic peptide

^a^Hazard ratio (95% confidence interval) for high- vs. low-MIF group

### MIF correlates with NPs and PASP

**Fig. 3:**
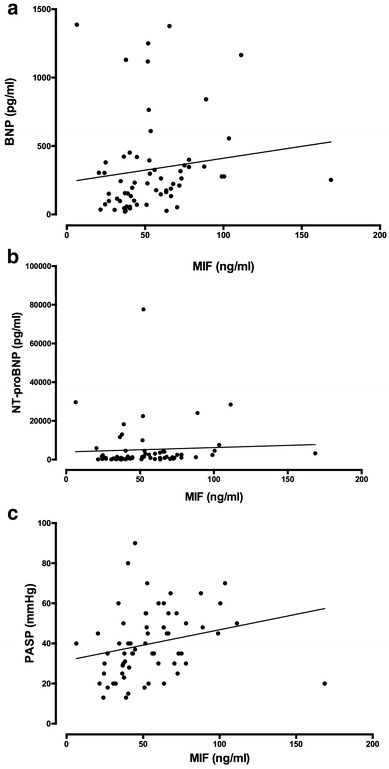
Scatter plots depicting the correlation of MIF with traditional biomarkers and clinical parameters. **a** BNP (r = 0.32, p = 0.011), **b** NT-proBNP (r = 0.28, p = 0.027), **c** PASP (r = 0.39, p = 0.0019)

To compare the analytical potential of MIF in HFpEF patients, we performed Spearman correlation to test the relationship between MIF and established disease markers (Fig. 3). MIF showed weak-to-moderate correlation with both NT-proBNP (*r* = 0.28, *p* = 0.027) and BNP (*r* = 0.32, *p* = 0.011) (Fig. 3a, b). Furthermore, MIF plasma levels significantly correlated with the estimated PASP (*r* = 0.39, *p* = 0.0019) (Fig. 3c). Thus, MIF showed a correlation to laboratory and clinical parameters of HFpEF in our cohort.

## Discussion

The key findings of the present study are as follows: (1) MIF correlates with symptoms of heart failure in HFpEF patients. (2) MIF shows a close correlation with surrogate parameter of pulmonary artery pressure. (3) MIF levels above the median are closely associated with the combined end-point of death or hospitalization after 180 days in HFpEF patients.

HFpEF is a disease of immense demographic and economic significance and to date there is no defined approach to monitor and to causally treat this condition [4]. As recently summarized in a scientific statement of the AHA, there is a need to further evaluate novel markers for guiding therapy and improve understanding of the pathophysiology and disease progression of heart failure [3]. Especially with regard to HFpEF, a biomarker should provide pathophysiological relevant information since established marker like the natriuretic peptides do not adequately mimic the disease course in these patients. The interpretation of partition values of natriuretic peptides is difficult in patients that show the typical characteristics of HFpEF like older age, obesity, atrial fibrillation, higher levels in women, and concomitant pulmonary disease. BNP and NT-proBNP levels must be interpreted having these limitations in mind. Even in patients with HFrEF, where the prognostic value of BNP and NT-proBNP is well established, it is completely unknown to date how these biomarkers need to be interpreted in future when patients are treated with the recently developed neprilysin inhibitors. Since neprilysin inhibitors increase BNP values and tend to decrease NT-proBNP levels, it is obvious that the diagnostic value of NPs will decrease with the growing number of patients treated with these new drugs. These circumstances substantiate the need for novel biomarkers in HFpEF.

Beyond this background, MIF seems to be a good candidate marker to be evaluated in HFpEF since it was shown to be a marker for oxidative stress, organ fibrosis, cell damage, atherosclerosis, lung disease, rheumatic diseases, sepsis [21], severe illness, and chronic kidney disease [12, 13, 18, 19, 22–25]. MIF is a pleiotropic upstream pro-inflammatory integral mediator of the innate immune system, stimulating the release of multiple cytokines, including tumor necrosis factor (TNF)-α which has been shown to activate matrix metalloproteinases and to be responsible for collagen degradation and progressive left ventricular dilation [26]. Elevated levels of inflammatory mediators have generally been identified in acute decompensated heart failure, as well as in patients with HFpEF. In our cohort, we could not detect any increase of inflammatory markers (CRP; WBC) and their correlation with MIF. Apart from inflammatory parameters, we found correlation between LDH and MIF as already demonstrated in resuscitation survivors [18]. This correlation might reflect the ubiquitous distribution of MIF throughout all types of cells within the organism. That goes along with the finding that both elevated MIF levels as well as LDH levels were found in the elderly patients. This might explain the increased amount of the classical “cell damage” marker LDH. But since MIF has been demonstrated to be a marker of cell damage, this might be an explanation.

The most robust correlation in our cohort could be demonstrated with NPs. Both BNP and NT-proBNP showed a close correlation with MIF and we further demonstrated that MIF has a comparable ROC with regard to symptoms of HFpEF as the NPs. This finding was paralleled by the fact that the high-MIF group had increased PASP compared to the low-MIF group. Whether this association is of causal relationship is speculative, but the current literature on the role of MIF in pulmonary hypertension, chronic lung disease, and idiopathic lung fibrosis draws a clear picture of MIF being elevated in these patients and contributes to the vascular remodeling processes in these diseases [27–29]. In experimental models, treatments with the MIF antagonist ISO-1 or anti-CD74 neutralizing antibodies partially reversed development of pulmonary hypertension in rats and substantially reduced inflammatory cell infiltration [28]. A recently developed MIF antagonist was even able to attenuate monocrotaline-induced pulmonary hypertension in rats [30]. It is well known that the presence of pulmonary hypertension is strongly associated with mortality in HFpEF patients [31, 32]. Since recent studies utilizing either echocardiography or right heart catheter indicated a pulmonary hypertension prevalence in a range between 36 and 83%, further studies should investigate whether MIF serves a role in the right heart pathophysiology in HFpEF [33].

Besides MIF plasma levels, we further performed 180-day follow-up and documented the time to first hospitalization or death from any reason in our cohort. Patients in the high-MIF group showed a weak correlation with the combined end-point. Despite the fact that Kaplan–Meier analyses must not be over-interpreted considering the small sample size, one must note that this correlation exists notwithstanding this limitation. Moreover, one must emphasize that the correlation between mortality and HFpEF is quite modest for most of the currently available biomarkers apart NPs (e.g., Galectin-3, GDF-15, sST2). For this reason, a multimarker strategy might overcome the limitations of single markers alone.

## Limitations

The current study exhibits some limitations that have to be addressed. The study population is quite small. That has to be taken into consideration when drawing conclusions from the current data. Nevertheless, this remains the first report on MIF in HFpEF and thus should be of hypothesis generating character. Despite the small sample size, there are some obvious correlations that should be in future studies with larger sample size.

## Conclusion

In this prospective cohort study, we analyzed the role of the cytokine MIF for the first time in patients with HFpEF. In our cohort, we demonstrate a clear association of MIF with symptoms, right heart loading conditions, and long-term outcome of these patients that was comparable with established biomarkers in heart failure. These first promising results on the role of MIF in HFpEF are encouraging for the conduction of further studies to evaluate MIF as part of a multimarker strategy in the prognostication of HFpEF.
